# Ang-(1–7) protects skeletal muscle function in aged mice

**DOI:** 10.1186/s12891-021-04693-9

**Published:** 2021-09-21

**Authors:** Ying Li, Jiao Song, Yangyang Jiang, Xue Yang, Li Cao, Chun Xiao, Suli Li, Birong Dong, Xiaoli Huang

**Affiliations:** 1 The Center of Gerontology and Geriatrics, National Clinical Research Center for Geriatrics, West China Hospital, Sichuan University, Sichuan, China; 2 Chengdu Koamy Biotechnology Co, Ltd, Chengdu, Sichuan China; 3 The Center of Gerontology and Geriatrics, National Clinical Research Center for Geriatrics,West China Hospital, Sichuan University, 37 Guoxuexiang, Sichuan providence 610041 Chengdu, P.R. China

**Keywords:** angiotensin-converting enzyme 2, skeletal muscle, angiotensin 1–7, aging

## Abstract

**Background:**

The angiotensin-converting enzyme 2 (ACE2)/angiotensin 1–7 (Ang-(1–7)) axis has been shown to protect against the age-associated decline in skeletal muscle function. Here, we investigated the protective effects of ACE2 in mitigating the age-associated decline of skeletal muscle function and to identify the potential underlying molecular mechanisms.

**Methods:**

We measured the expression levels of Ang-(1–7) in C57BL/6J mice of different ages and correlated these levels with measures of skeletal muscle function. We also investigated the expression of myocyte enhancer factor 2 A (MEF2A) in ACE2 knockout (ACE2KO) mice and its relationship with muscle function. We then treated aged ACE2KO mice for four weeks with Ang-(1–7) and characterized the levels of MEF2A and skeletal muscle function before and after treatment. We assessed the impact of Ang-(1–7) on the growth and differentiation of C2C12 cells in vitro and assessed changes in expression of the glucose transporter type 4 (Glut4).

**Results:**

Aged mice showed reduced skeletal muscle function and levels of Ang-(1–7) expression in comparison to young and middle-aged mice. In ACE2KO mice, skeletal muscle function and MEF2A protein expression were significantly lower than in age-matched wild-type (WT) mice. After one month of Ang-(1–7) treatment, skeletal muscle function in the aged ACE2KO mice improved, while MEF2A protein expression was similar to that in the untreated group. In C2C12 cells, Ang-(1–7) was shown to promote along with the upregulated expression of Glut4.

**Conclusions:**

The ACE2/ Ang-(1–7) axis has a protective function in skeletal muscle and administration of exogenous Ang-(1–7) can delay the age-related decline in the function of skeletal muscle.

## Introduction

The process of aging is correlated with pronounced decreases in skeletal muscle mass, reduced muscular function and a general decline in physical fitness. In the clinic, these manifestations are commonly associated with sarcopenia and frailty [1]. Selective androgen agonists can be prescribed to partially increase muscle mass; however, these agents fail to improve muscle function. Although myostatin-neutralizing antibody and activin IIB receptor blockers can significantly increase muscle mass and partially improve muscle function, their clinical benefits are unclear [2, 3]. The identification of safe and clinically effective drugs capable of managing the age-related loss of muscle function and mass represents a significant challenge with a high potential for clinical impact.

The Renin-angiotensin system (RAS), a peptidergic signaling pathway, is widely expressed in the cardiovascular system, kidneys, and lungs. The association between RAS and the angiotensin-converting enzyme (ACE)-angiotensin II (Ang II) axis is well documented. However, the angiotensin-converting enzyme 2 (ACE2) - angiotensin 1–7 (Ang-(1–7)) pathway has been reported to compete with ACE/Ang II pathway [4].

The main pathological features of sarcopenia are directly related to the age-related decline in the mass and structure of skeletal muscles and the ACE/Ang II pathway has been found to be closely associated with skeletal muscle loss [5]. Ang II, through binding to the angiotensin II type 1 receptor (AT1R), reduces microvascular perfusion, glucose uptake, and insulin sensitivity in mouse muscle fibers [6, 7]. Attempts have been made to improve skeletal muscle function and delay age-related muscle atrophy by pharmacological antagonism of the ACE-Ang II-AT1R pathways using AT1R antagonists or ACE inhibitors (ACEIs). However, randomized controlled clinical trials have so far demonstrated unsatisfactory results. Shrikrishna and colleagues showed that ACEI did not significantly improve lower extremity skeletal muscle function in elderly patients with severe obstructive pulmonary illness [8]. Similarly, the TRAIN trial demonstrated that ACEI had no significant effects on the activity capacity of elderly patients [9].

The ACE2 - Ang-(1–7) pathway is the protective arm of RAS. Several studies have reported that Ang-(1–7) attenuates muscle dysfunction in animal models. Acuña et al. [10] found that Ang-(1–7) decreased fibrosis and improved muscle function in Duchenne muscular dystrophy mice. In chronic liver disease mice, Ang-(1–7) prevented declining muscle strength and function, reducing fatigue [11]. Thus, activation of the ACE2 - Ang-(1–7) pathway might contribute to the improvement of muscle decline in age-associated muscular disorders. The main aim of the current research was to investigate the role of the ACE2/Ang-(1–7) pathway on age-related skeletal function decline. Additionally, we attempted to clarify the possible underlying molecular mechanisms of this protective action.

## Methods

### Experimental design

C57BL/6J mice of different ages were used for the in vivo experiments. An ACE2-KO mouse model (C57BL/6J background) was also used for functional studies. All mice were bought from the Laboratory Animal Research Institute of the Chinese Academy of Medical Sciences. In vitro assays were performed using the C2C12 mouse myoblast cell line. Experiments were performed to determine skeletal muscle function and the expression of Ang (1–7) and Ang II in old (20 months), middle-aged (12 months), and young (3 months) C57BL/6J mice. Further, the skeletal muscle function and the expression of myocyte enhancer factor 2 A (MEF2A) were determined in young (4 months) and old (16 months) ACE2-KO mice and their wild-type age-matched controls.

Old (16 months) ACE2-KO mice received Ang-(1–7) (APEXBio, Houston, TX, USA, product No. A1041) administered through osmotic minipumps (Alzet-Durect, Cupertino, CA, USA) with 400 ng/kg/min of for four weeks. Skeletal muscle function and the expression of MEF2A were compared before and after treatment. In the control group, the ACE2-KO mice were received 0.9 % normal saline. Each group had five mice. C2C12 cells (from the cell bank of the Chinese Academy of Sciences) were cultured in Dulbecco’s Modified Eagle Medium (DMEM) supplemented with 10 % fetal bovine serum (Gibco, Grand Island, NY, USA). To induce cell differentiation, cells were cultured for 48 h in DMEM containing 2 % horse serum (Gibco, Grand Island, NY, USA), supplemented with 10^− 8^ mol/L of Ang-(1–7) or the same volume of PBS. After incubation, the cellular differentiation levels and the expression of glucose transporter type 4 (Glut4), MEF2A, myosin heavy chain (MHC), and creatine kinase, muscle (CKM) proteins were evaluated. All in vivo protocols were approved by the Animal Committee of West China Hospital, Sichuan University.

### Testing of skeletal muscle function

Skeletal muscle function was evaluated by measuring the grip forelimb strength and the number of falls in a treadmill test. The grip strength of the mouse forelimb was measured using a tester of grip strength (YLS-13 A, Jinan Yiyan Technology Development Co., Ltd, Jinan, China.). For each mouse, five measurements were performed at 5-seconds intervals and the average value was recorded. The treadmill test used an experimental animal treadmill (ZH-PT, Shanghai Kehuai Instrument Co., Ltd, Shanghai, China., Shanghai, China) with the track speed set at 9 m/min. The number of falls from the track within 5 min was recorded. For each mouse, measurements were recorded in triplicate and performed at 5-minute intervals. The average number of falls was recorded.

### Preparation of mouse skeletal muscle

Mice were anesthetized by using pentobarbital (45 mg/kg) and the gastrocnemius and soleus muscles were removed and frozen in liquid nitrogen for subsequent analyses.

### Determination of the levels of Ang-(1–7) and Ang II in mouse skeletal muscles

Frozen skeletal muscle samples (30 mg) were thawed and homogenized in a cold mixture of methanol (500 µl) and phosphate buffer (500 µl, pH 7.4, 50 mM), and then incubated at 4 ℃ for 1 h and centrifuged at 100,000 g for 20 min. The concentrations of Ang II and Ang-(1–7) were measured in the supernatants using ELISA kits (Cloud-Clone Corp., Houston, TX, USA) Cat. No. CEA005Mu (Ang II) and CES085Mi (Ang-(1–7)) according to the manufacturers’ instructions. The levels of Ang II and Ang-(1–7) were measured in muscle samples obtained from five mice in each experimental group.

### Determination of MEF2A protein levels in mouse skeletal muscle

The expression of MEF2A was determined by Western blotting. The absorbance of each protein band was determined using Image-Pro Plus 6.0 software.

### C2C12 cell culture and differentiation

C2C12 cells were cultured in DMEM containing 2 % horse serum. To analyze cell differentiation, Ang-(1–7) was added and the cells were incubated at 37℃ for an additional 48 h. PBS was added to the control cultures. The fusion index was calculated as the ratio of the number of nuclei in myocytes with two or more nuclei versus the total number of nuclei.

### Protein expression in differentiating C2C12 cells

The expression of MHC, creatine kinase, muscle CKM, Glut4, and MEF2A proteins was determined by Western blotting in C2C12 cells using three independent experiments. Following the evaluation of differentiation, the cells were collected, rinsed with PBS, incubated on ice in RIPA lysis buffer, and centrifuged at 100,000 g for 20 min at 4 °C. The total protein concentration in the supernatants was quantified by the BCA method. Proteins (20 µg/lane) were separated on SDS-PAGE, transferred to nitrocellulose membranes, and blocked as described above. Primary antibodies against MHC (Proteintech, Rosemont, IL, USA, Cat. No. 10799-1-AP), CKM (Proteintech, Cat. No. 18712-1-AP), Glut4 (Proteintech, Rosemont, IL, USA, Cat. No. 21048-1-AP), and MEF2A (Proteintech, Rosemont, IL, USA, Cat. No. 12382-1-AP) were incubated overnight at 4 °C. The blots were then probed with secondary antibodies and visualized using a chemiluminescence detection kit. Protein expression was normalized to that of GAPDH (AC036; ABclonal). The absorbance of each protein band was determined using the Image-Pro Plus 6.0 software.

## Statistical methods

Data were expressed as means ± SD (five animals per group, three independent experiments) and analyzed utilizing the SPSS 20.0 computer program package. A t-test was utilized for comparison between two groups. One-way ANOVA or the Kruskal-Wallis test were used for comparison between several groups. A *P*-value threshold of 0.05 was considered statistically significant.

## Results

**Fig. 1:**
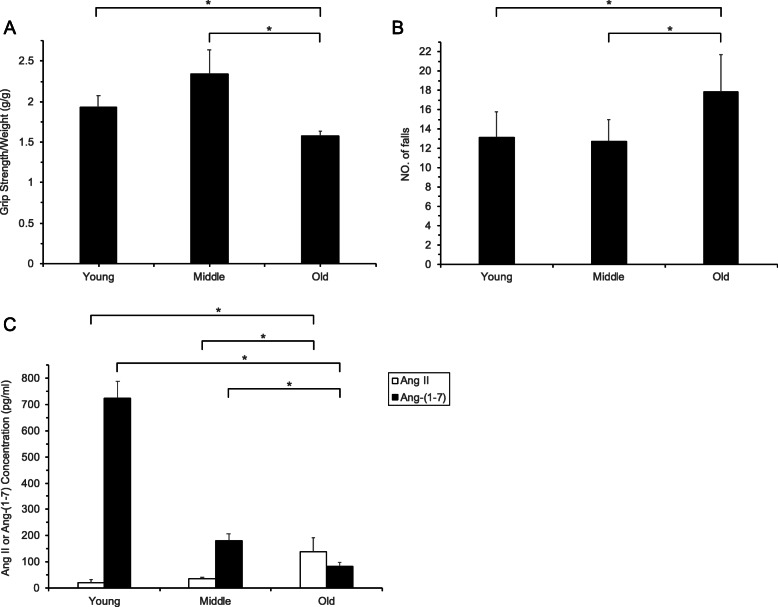
(**A**) grip strength, (**B**) numbers of falls, and (**C**) Ang-(1–7) and Ang II expression in the treadmill test in mice of different ages. * *P* < 0.05, determined by the ANOVA with Least Significance Difference test or Kruskal-Wallis one way ANOVA test (*n* = 5 mice per group).

The grip strength of old mice was observed to be decreased compared to young and middle-aged mice (young: 1.93 ± 0.14 g/g, middle-aged: 2.34 ± 0.29 g/g, old: 1.58 ± 0.06 g/g, all *P* < 0.05) (Fig. 1 A). In the treadmill test, the number of falls in old mice (17.86 ± 3.85) was higher than in the young (13.14 ± 2.60) and middle-aged (12.71 ± 2.29) mice (*P* < 0.05) (Fig. 1 B). The levels of Ang-(1–7) in the skeletal muscles of old mice were found to be lower compared to the young and middle-aged mice. The levels of Ang II were greater than in young and middle-aged mice compared to old mice (*P* < 0.05 in all cases) (Fig. 1 C). These data indicated that the function of mouse skeletal muscle and the expression of Ang-(1–7) were both reduced with aging. In contrast, the expression of Ang II increased with aging in mice.

**Fig. 2:**
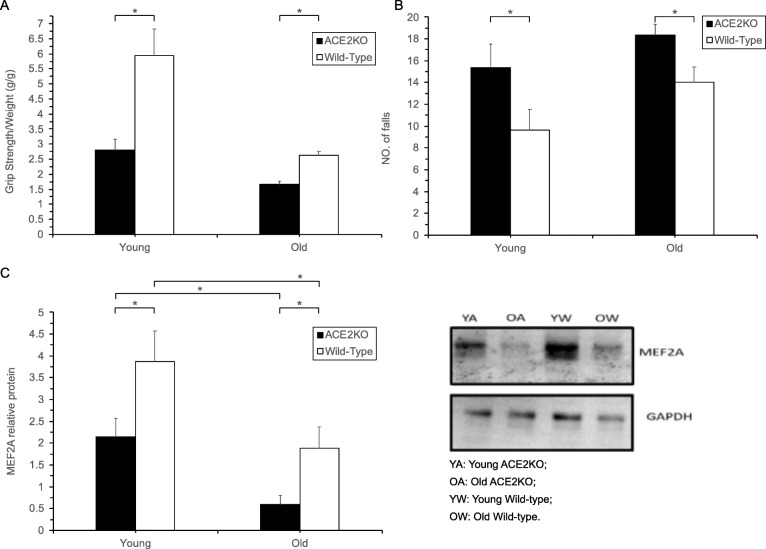
(**A**) grip strength, (**B**) numbers of falls, and (**C**) MEF2A expression in ACE2-KO and WT mice. *P < 0.05, determined by t-test (*n* = 5 mice per group).

We compared the motor abilities of ACE2-KO and wild-type (WT) mice at various ages and found that the grip strength of ACE2-KO mice was considerably lower than age-matched WT animals (*P* < 0.05). Moreover, the grip strength of young ACE2-KO mice was similar to that of old WT mice (2.80 ± 0.34 g/g vs. 2.63 ± 0.13 g/g, *P* > 0.05) (Fig. 2 A).

In the treadmill experiments, the number of falls was significantly higher in the ACE2-KO mice in comparison to the age-matched WT animals (*P* < 0.05). The number of treadmill falls in young ACE2-KO was comparable to that in old WT mice (15.33 ± 2.16 vs. 14.00 ± 1.41, *P* > 0.05) (Fig. 2 B).

The changes in the levels of MEF2A protein expression in the mouse skeletal muscles at different ages indicated that the level of MEF2A in both WT and ACE2-KO mice decreased with age (both P < 0.05). However, the expression of MEF2A was lower in ACE2-KO than in WT mice (*P* < 0.05) (Fig. 2 C).

**Fig. 3:**
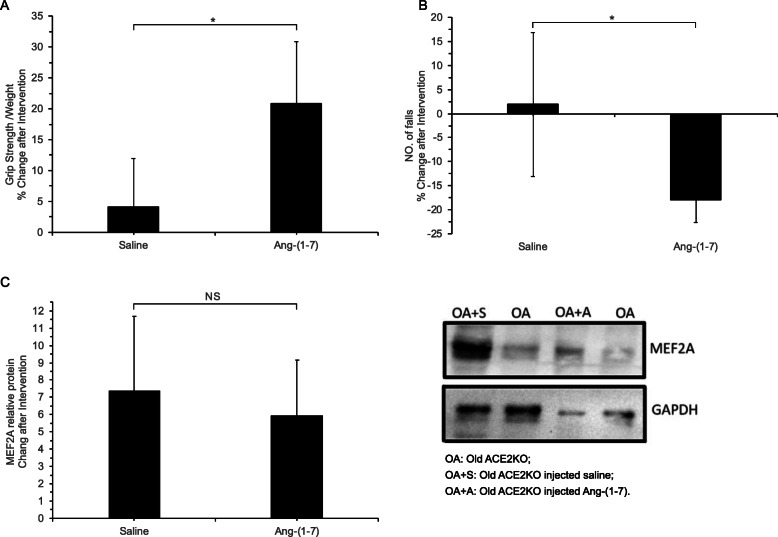
(**A**) Changes in grip strength, (**B**) numbers of falls, and (**C**) MEF2A expression after Ang-(1–7) intervention in older ACE2-KO mice. * *P* < 0.05, determined by t-test (*n* = 5 mice per group).

After four weeks of Ang-(1–7) injections, the grip strength of aged ACE2-KO increased (*P* < 0.05) (Fig. 3 A), and the number of falls in the treadmill test decreased (*P* < 0.05) (Fig. 3 B). However, the expression of MEF2A protein in skeletal muscle remained similar to that in the normal saline group (*P* > 0.05) (Fig. 3 C). Both skeletal muscle function and MEF2A protein expression decreased in aged ACE2-KO mice. The administration of Ang-(1–7) in ACE2-KO mice increased their motor ability without producing a significant change in the MEF2A protein level.

**Fig. 4:**
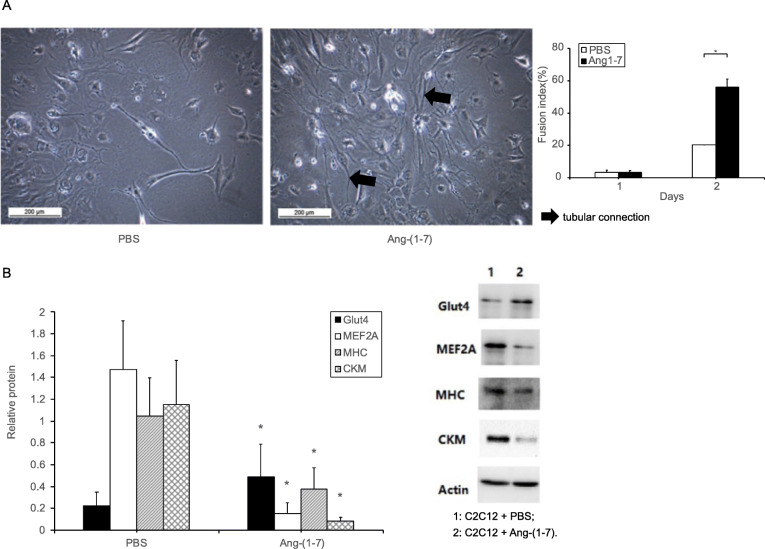
(**A**) Differentiation and (**B**) Glut4, MEF2A, MHC, and CKM expression of C2C12 cells after Ang-(1–7) intervention. * *P* < 0.05, determined by t-test between intervention and control groups (*n* = 3 independent experiments per group).

The influences of Ang-(1–7) on the differentiation of C2C12 cells in vitro were evaluated. After the exposure to Ang-(1–7) for 48 h, C2C12 cells formed tentacle-like extensions that had fibroblast-like morphologies. Importantly, tubular connections between cells were formed (Fig. 4 A). The fusion index of the Ang-(1–7) intervention group was higher than that of the control group. Western blotting showed that Ang-(1–7) treatment upregulated the expression of Glut4 protein but down-regulated the levels of MEF2A, MHC, and CKM (Fig. 4 B). Treatment with Ang-(1–7) promoted the differentiation and growth of C2C12 cells and enhanced the expression of Glut4.

## Discussion

In this study, it was found that with increasing age, both the skeletal muscle function and the tissue levels of Ang-(1–7) decreased in mice. The skeletal muscle function in old ACE2-KO mice was reduced and the motor ability of young ACE2-KO mice was equivalent to that of old wild-type mice. However, the motor function of aged ACE2-KO mice increased after Ang-(1–7) treatment. We investigated the possible underlying mechanisms of these effects and found that Ang-(1–7) treatment resulted in the upregulated expression of Glut4 while not affecting the expression of MEF2A. These results indicate that Ang-(1–7) enhances skeletal muscle motor function in aging mice by promoting cellular glucose metabolism.

Our experiments demonstrated that the older mice had reduced grip strength and experienced a higher number of falls compared to young mice in the treadmill test. The skeletal muscles of aged animals showed decreased levels of Ang-(1–7) and higher levels of Ang II compared to young mice. Previous findings have shown that the ACE/Ang II axis is correlated with skeletal muscle insulin resistance, atrophy and fibrosis and antagonizes the ACE2/Ang-(1–7) axis [12, 13]. The ACE/Ang II axis reduces microvascular perfusion, glucose uptake, and the insulin sensitivity of insulin during skeletal muscle atrophy [6, 7].

In contrast, ACE2/Ang-(1–7) axis is beneficial to skeletal muscles as it acts to improve insulin resistance and prevent fibrosis [14, 15]. The current study showed that the decline in skeletal muscle function in aged mice was accompanied by a decrease in the tissue concentration of Ang-(1–7) and an enhancement in the concentration of Ang II. These findings suggest that as mice age, an imbalance in the RAS system may develop in skeletal muscles. These changes cause decreased performance of the protective ACE2/Ang-(1–7) axis and an increase in the deleterious activity of the ACE/Ang II pathway.

Further analysis showed that at any age, the skeletal muscle performance in ACE2-KO mice was worse than in WT animals, and the muscle performance in young ACE2-KO mice was similar to that in aged WT mice. Continuous injection of Ang-(1–7) in old ACE2-KO mice increased motor capacity which was consistent with the findings of Takeshita and Hikari [16, 17]. The deletion of the *ACE2* gene accelerated the age-associated damage to skeletal muscle function in mice whilst the administration of Ang-(1–7) alleviated this type of damage.

In animal models of heart failure, ACE2 improves cardiac function and ventricular remodeling by the production of Ang-(1–7) resulting in improved survival [18]. In another study, the treatment of ACE2-KO mice by exogenous Ang-(1–7) reduced cardiac lipid toxicity and atherosclerosis caused by a high-fat diet and prevented the development of heart failure [19]. Similarly, in skeletal muscle, Ang-(1–7) has been demonstrated to enhance microvascular perfusion and glucose uptake and to improve insulin resistance [14, 20].

In a mouse model of Duchenne muscular dystrophy, the upregulation of ACE2 expression or the administration of Ang-(1–7) were observed to reduce the skeletal muscle fibrosis and to ameliorate skeletal muscle structure and function [10, 21, 22]. Our data are also supported by the work from the Yamamoto laboratory that showed that ACE2 deficiency accelerated the age-related loss of skeletal muscle mass and functional decline. However, the Yamamoto group found that the protective effect of ACE2 on skeletal muscles may be achieved through the non-Ang-(1–7)-MAS pathway [17, 23]. Furthermore, apart from the ACE2/Ang-(1–7) pathway, ACE2 is also involved in other biological pathways including the ACE2/Apelin pathway that acts to reverse sarcopenia and age-related muscle loss and functional decline [24]. Further in vivo studies are required to identify the pathways through which ACE2 achieves its protective effect on skeletal muscle during aging.

Although ACE2 may exert its biological effects in ACE2-KO mice through a variety of pathways, the delivery of exogenous Ang-(1–7) was shown to slow the process of age-associated skeletal muscle decline resulting in increased muscle function. We then explored the specific mechanism underlying the impact of exogenous Ang-(1–7) in vitro. After the exposure of C2C12 cells to Ang-(1–7), the expression of Glut4 increased and the expression of MEF2A decreased. Takeda and coworkers [25] reported that the expression of Glut4 protein was reduced in the skeletal muscle of ACE2-KO mice. After treatment with Ang-(1–7), the expression of Glut4 increased in ACE2-KO mice. In addition, they found that Ang-(1–7) increased insulin-stimulated glucose uptake both in vivo and in vitro. Glut4 is responsible for glucose transport in skeletal muscle cells and regulates glucose homeostasis to reduce insulin resistance [26, 27]. Regular exercise can improve the expression of Glut4 in the muscles thereby increasing glucose uptake capacity and improving muscle function [26]. The results presented in the current study also suggested that Ang-(1–7) might promote glucose metabolism in muscle cells.

There are some limitations in the current study. We did not measure the Glut4 levels in skeletal muscle of ACE2-KO mice. We also did not perform a glucose tolerance test in mice or measure glucose uptake in the C2C12 cells. Previous studies found that Ang-(1–7) enhanced the glucose tolerance and improved insulin resistance in rodents [25, 28, 29]. In vitro, Ang-(1–7) treatment enhanced insulin mediated glucose uptake [25]. These studies focused on the relationship between Ang-(1–7) and metabolic regulation, but did not investigated skeletal muscle aging. Thus, further research will be required to investigate the precise involvement of glucose metabolism in the Ang-(1–7)-induced protective mechanism in skeletal muscle aging.

## Conclusions

An imbalance of the RAS system may be responsible for the age-associated decline in skeletal muscle function. The ACE2/Ang-(1–7) axis has a protective role in skeletal muscles and the administration of exogenous Ang-(1–7) can prevent the deterioration in skeletal muscle function. The possible mechanism is that Ang-(1–7) improves the glucose metabolism in muscle cells. This study provides new perspectives for the treatment of age-related sarcopenia with Ang-(1–7).

## Data Availability

The datasets used and/or analyzed during the current study are available from the corresponding author on reasonable request.

## Abbreviations

ACE: angiotensin-converting enzyme
Ang: angiotensin
MEF2A: myocyte enhancer factor 2 A
ACE2KO: ACE2knockout
Glut4: glucose transporter type 4
RAS: Renin-angiotensin system
ACEI: Angiotensin converting enzyme inhibitor
MHC: myosin heavy chain
CKM: creatine kinase muscle
